# Long-term outcome of infective endocarditis: A study on patients surviving over one year after the initial episode treated in a Finnish teaching hospital during 25 years

**DOI:** 10.1186/1471-2334-8-49

**Published:** 2008-04-17

**Authors:** Maija Heiro, Hans Helenius, Saija Hurme, Timo Savunen, Kaj Metsärinne, Erik Engblom, Jukka Nikoskelainen, Pirkko Kotilainen

**Affiliations:** 1 Department of Medicine, Turku University Hospital, Turku, Finland; 2 Department of Biostatistics, University of Turku, Turku, Finland; 3 Department of Surgery, Turku University Hospital, Turku, Finland

## Abstract

**Background:**

Only a few previous studies have focused on the long-term prognosis of the patients with infective endocarditis (IE). Our purpose was to delineate factors potentially associated with the long-term outcome of IE, recurrences of IE and requirement for late valve surgery.

**Methods:**

A total of 326 episodes of IE in 303 patients were treated during 1980–2004 in the Turku University Hospital. We evaluated the long-term outcome and requirement for late valve surgery for 243 of these episodes in 226 patients who survived longer than 1 year after the initial admission. Factors associated with recurrences were analysed both for the 1-year survivors and for all 303 patients.

**Results:**

The mean (SD) follow-up time for the 1-year survivors was 11.5 (7.3) years (range 25 days to 25.5 years). The overall survival was 95%, 82%, 66%, 51% and 45% at 2, 5, 10, 15 and 20 years. In age and sex adjusted multivariate analyses, significant predictors for long-term overall mortality were heart failure within 3 months of admission (HR 1.97, 95% CI 1.27 to 3.06; p = 0.003) and collagen disease (HR 2.54, 95% CI 1.25 to 5.19; p = 0.010) or alcohol abuse (HR 2.39, 95% CI 1.30 to 4.40; p = 0.005) as underlying conditions, while early surgery was significantly associated with lower overall mortality rates (HR 0.31, 95% CI 0.17 to 0.58; p < 0.001). Heart failure was also significantly associated with the long-term cardiac mortality (p = 0.032). Of all 303 patients, 20 had more than 1 disease episode. Chronic dialysis (p = 0.002), intravenous drug use (p = 0.002) and diabetes (p = 0.015) were significant risk factors for recurrent episodes of IE, but when analysed separately for the 1-year survivors, only chronic dialysis remained significant (p = 0.017). Recurrences and late valve surgery did not confer a poor prognosis.

**Conclusion:**

Heart failure during the index episode of IE was the complication, which significantly predicted a poor long-term outcome. Patients who underwent surgery during the initial hospitalisation for IE faired significantly better than those who did not.

## Background

The short-term outcome of infective endocarditis (IE) has been examined in several previous studies from the 1990's and 2000's [1-4], whereas only a small number of studies have addressed the long-term prognosis of the patients with IE. Moreover, most of the studies focusing on the long-term prognosis have involved only certain subgroups of patients, e.g., those with late prosthetic valve IE [5], IE with perivalvular abscess [6], surgically treated IE associated with intravenous drug use (IVDU) [7], or native valve IE in non-addicts [8], as well as of those undergone surgery for defined indications, e.g. aortic valve IE [9], mitral valve IE [10], or native or prosthetic valve IE [11]. Instead, the long-term outcome of IE in large series of unselected patients with IE has been the focus of rather few studies [12-15].

We have previously analysed the short-term and 1-year clinical outcome of 303 patients with 326 episodes of IE treated in our hospital during the years 1980–2004 [16]. In the present study, we set out to analyse the long-term clinical outcome of 243 of these episodes in 226 patients who survived longer than 1 year after the initial admission for IE. Our purpose was to delineate the clinical characteristics of the patients during the index hospitalisation for IE, which might be predictive of the long-term outcome of IE. One important issue involved the impact of early surgery on the long-term survival of the patients. We also evaluated risk factors potentially associated with the development of recurrences of IE and the requirement for late valve surgery.

## Methods

Between 1980 and 2004, 303 patients with 326 episodes of IE were treated in the Turku University Hospital, Turku, Finland. We evaluated the long-term outcome of 226 of these patients who survived longer than 1 year after the initial admission. The hospital is a 1000-bed teaching facility with a cardiothoracic surgical department, serving as a tertiary referral centre for the southwestern part of the country, and as a primary care facility for infectious diseases for a region of about 200.000 inhabitants.

For each patient, data on age, sex, underlying diseases, causative agents of IE, affected valves, predisposing cardiac conditions and echocardiographic findings, as well as the development of complications and the need for valve surgery were collected by us for our previous study focusing on the changes of the clinical characteristics of IE during the 25-year study period [19]. In addition, serum C-reactive protein (CRP) values, erythrocyte sedimentation rates (ESR) and white blood cell (WBC) counts on admission were registered previously [16,20], as were the results of the polymerase chain reaction analyses from the removed valve tissue for those patients who underwent surgery during 1994–2005 [21]. In the present study, these data were used to analyse the association between various patient and disease characteristics and the long-term outcome of the patients after an episode of IE. The survival and causes of death were assessed based on data obtained from the National Population Registry, in which all deaths and causes of deaths of the Finnish residents are registered. The patients were also analysed for factors associated with recurrent episodes of IE and with requirement for late valve surgery. Data regarding the recurrences and late valve surgery were obtained from the hospital records of the patients.

The follow-up period for the outcome was from the admission to death, or if alive, up to February 2006.

### Definitions

Long-term outcome was defined as the outcome > 1 year after the admission for the index episode of IE. Early surgery was defined as surgery during the index hospitalisation, and late surgery was defined as surgery > 1 year after the initial admission. A recurrence was defined as a new episode of IE caused by a different microorganism or by a microorganism of the same genus or species of the previous episode of IE after a period of more than 6 months. A relapse was defined as a new episode of IE caused by a microorganism of the same genus and species within 6 months after discharge.

The study was approved by the Institutional Committee on human research.

### Statistical analysis

Four different end-points were analysed in the study: long-term overall mortality, long-term cardiac mortality, late valve surgery, and recurrent IE. The associations between the clinical characteristics and end-points were studied separately using survival analysis. The cumulative percentages for survival during different time periods were estimated using the Kaplan-Meier technique. Differences in cumulative percentages between groups were tested using log-rank test. Data regarding the long-term overall outcome, cardiac mortality, and late valve surgery were analysed for the 1-years survivors, and data regarding the repeated episodes of IE both for the 1-year survivors and for all 303 patients.

Early surgery, late surgery and recurrent IE were analysed as time-dependent covariates applying Cox's regression models. Differences between groups were quantified by hazard ratios and ninety-five percent confidence intervals.

Multivariate analyses of end-points were carried out using the Cox regression model with early surgery as a time-dependent covariate. Stepwise method was used for model selection. The results of multivariate analysis were adjusted for age and sex and differences between groups were quantified by hazard ratios and ninety-five percent confidence intervals. Except for the laboratory parameters and indications for surgery, all of the clinical characteristics presented in Table 1 were included in multivariate analyses.

**Table 1:** Association between characteristics of infective endocarditis (IE) and survival (global and in different subgroups) at 2, 5, 10, 15, and 20 years after the admission in 243 episodes of IE in 226 patients who survived over 1 year after the initial episode of IE

	**Number of IE episodes in patients surviving 1 year**	**Survival (%) at different points of follow-up**					**p value**
			
		**2 years**	**5 years**	**10 years**	**15 years**	**20 years**	
**Global survival**	243	95	82	66	51	45	
**Gender**							
Male	174	97	83	68	50	45	0.567
Female	69	91	80	59	51	46	
**Age**							
18–64 years	172	99	90	76	60	54	< 0.001
≥ 65 years	71	87	65	38	25	25	
**Affected valves**							
Aortic	85	95	85	63	52	46	0.171*
Mitral valve	77	95	80	58	40	40	
Tricuspid valve	12	100	100	100	NA†	NA	
Two native valves	17	100	100	91	76	76	
Prosthetic valve(s)	52	94	74	67	52	43	
**Causative agents of IE**							
*Staphylococcus aureus*	53	98	86	77	57	57	0.183*
Coagulase-negative staphylococci	22	86	58	41	41	41	
Viridans streptococci	50	96	91	77	56	50	
*Enterococcus faecalis*	18	83	83	67	28	28	
*Streptococcus pneumoniae*	5	100	100	53	53	53	
Other	22	95	91	66	60	30	
Negative	73	99	78	61	46	46	
**Predisposing cardiac condition**							
Acquired valvular disease	49	90	70	53	41	36	0.019
Prosthetic valves	55	95	75	66	52	43	
Bicuspid aortic valve	26	100	100	94	81	81	
Mitral valve prolapse	30	97	93	74	62	62	
Congenital heart disease	8	100	100	33	33	NA	
No underlying cardiac condition	75	97	83	63	39	39	
**Echocardiogarphic findings**							
**Major criteria**							
Yes	158	96	85	68	53	49	0.089
No	85	95	78	61	46	39	
**Vegetation**							0.162
Yes	139	95	85	68	52	49	
No	104	96	80	62	49	41	
**Underlying conditions**							
**Diabetes**							
Yes	21	95	95	77	58	29	0.307
No	222	95	81	64	49	45	
**Dialysis**							
Yes	5	60	40	NA	NA	NA	0.018
No	238	96	83	66	51	46	
**Collagen disease**							
Yes	14	93	68	24	24	24	0.026
No	229	96	83	68	52	46	
**Malignancy**							
Yes	11	82	62	31	NA	NA	0.004
No	232	96	83	67	53	47	
**Intravenous drug use**							
Yes	19	100	100	NA	NA	NA	0.049
No	224	95	81	64	50	44	
**Alcohol abuse**							
Yes	22	91	67	47	35	NA	0.029
No	221	96	84	68	53	47	
**Heart failure**††							0.047
Yes	108	93	75	55	46	44	
No	135	98	89	75	55	46	
**Neurological complications††**							
Yes	60	95	78	66	52	52	0.766
No	183	96	84	65	50	43	
**Peripheral emboli††**							
Yes	69	97	83	60	50	50	0.570
No	174	95	82	67	51	43	
**Early surgery (in-hospital)**							
Yes	67	99	89	82	76	73	< 0.001
No	176	94	80	59	41	34	
**Mode of treatment**††							
Surgical	72	99	90	82	74	70	< 0.001
Conservative	171	94	79	59	41	33	
**Indications for in-hospital surgery**							
Congestive heart failure	35	97	85	72	61	55	0.002*
Valvular regurgitation without heart failure	18	100	94	94	94	94	
Dehiscence of prosthetic valve without heart failure	8	100	88	88	88	88	
Repeated emboli	3	100	100	100	100	100	
Intractable infection	3	100	100	100	100	100	
**Erythrocyte sedimentation‡,§**							
≥ 50 mm/h	75	93	76	55	40	34	0.018
< 50 mm/h	74	97	89	77	56	53	
**White blood cell count‡**,**§S**							
≥ 10 × 10^9^/l	119	97	83	63	41	41	0.142
< 10 × 10^9^/l	98	94	82	67	61	53	
**Serum C-reactive protein‡**,¶							
≥ 100 mg/l	100	96	83	62	48	48	0.927
< 100 mg/l	105	94	80	68	51	43	
**Serum creatinine value‡¶¶**							< 0.001
≥ 100 μmol/l	76	95	77	53	27	14	
< 100 μmol/l	142	97	87	71	61	59	

*p values for overall group differences; †NA = non-applicable, the follow-up did not reach the time point for any patient of the group; ††within 3 months of admission; ‡on admission; §data available for 149 episodes; §Sdata available for 217 episodes; ¶data available for 205 episodes; ¶¶ data available for 218 episodes

P-values less than 0.05 were considered as statistically significant. Statistical computations were carried out using SAS^® ^release 9.1/2005.

## Results

The mean (SD) follow-up time for the 303 patients with 326 episodes of IE treated between 1980–2004 was 12.2 (7.4) years (range 1.0 to 25.5 years). The median survival was 5.0 years for all patients, and the range of survival for those who died during the follow-up was 4 days to 23 years. The overall survival for all 303 patients was 75%, 72%, 62%, 49%, 38% and 34 % at 1, 2, 5, 10, 15, and 20 years. We included in this study the 226 patients, who survived longer than 1 year after the initial admission for IE. These patients had altogether 243 episodes of IE, of which 155 were designated as definite IE and the rest as possible IE by the Duke diagnostic criteria [17]. A detailed diagnostic classification has been previously published by us for 142 of these episodes [18].

There were 174 episodes in men and 69 episodes in women. The mean age (SD) of the patients was 52.4 (17.1) years (range 18 to 87 years). The mean (SD) follow-up time for the l-year survivors was 11.5 (7.3) years (range 25 days to 25.5 years), and the 25% percentile of the follow-up time was 4.8 years and the 75% percentile of the follow-up time was 17.8 years. The median survival was 7.1 years, and the range of survival for those who died during the follow-up was 4 days to 22.3 years. Of these patients, 67 underwent in-hospital valve surgery and 72 were treated both surgically and conservatively within 3 months of the admission. The proportions of various clinical characteristics (e.g. causative agents of IE, affected valves, predisposing cardiac conditions, development of complications) are presented in Tables 1 and 2. A total of 94 patients died during the follow-up. The causes of death were: recurrent IE (n = 1), sequelae of IE (n = 27), coronary heart disease (n = 18), malignancy (n = 16), infection other than IE (n = 14), stroke (n = 6), other or unknown cause (n = 12). Of the 27 patients who died of sequelae of IE, the cause of death was established to be heart failure in 18 patients and sudden death due to arrhythmia in 9 patients.

**Table 2:** Association between characteristics of infective endocarditis (IE) and cardiac mortality at 2, 5, 10, 15, and 20 years after the admission in 243 episodes of IE in 226 patients who survived over 1 year after the initial episode of IE

	**Number of IE episodes in patients surviving 1 year**	**Survival (%) at different points of follow-up**					**p value**
			
		**2 years**	**5 years**	**10 years**	**15 years**	**20 years**	
**Cardiac mortality**	243	3	10	16	25	29	
**Gender**							
Male	174	2	9	15	26	31	0.801
Female	69	6	11	20	24	24	
**Age**							
18–64 years	172	1	5	11	22	26	0.002
≥ 65 years	71	10	21	32	32	32	
**Affected valves**							
Aortic	85	4	9	18	24	29	0.416*
Mitral valve	77	4	9	20	34	34	
Tricuspid valve	12	0	0	0	NA	NA	
Two native valves	17	0	0	0	17	17	
Prosthetic valve(s)	52	4	17	17	24	29	
**Causative agents of IE**							
*Staphylococcus aureus*	53	2	4	4	4	4	0.032*
Coagulase-negative staphylococci	22	10	20	30	30	30	
Viridans streptococci	50	2	2	9	34	40	
*Enterococcus faecalis*	18	17	17	25	63	63	
*Streptococcus pneumoniae*	5	0	0	0	0	0	
Other	22	0	0	16	16	28	
Negative	73	1	17	22	25	25	
**Predisposing cardiac condition**							
Acquired valvular disease	49	8	19	29	41	41	0.015*
Prosthetic valves	55	4	16	16	23	28	
Bicuspid aortic valve	26	0	0	0	0	0	
Mitral valve prolapse	30	3	3	15	29	29	
Congenital heart disease	8	0	0	38	38	NA	
No underlying cardiac condition	75	1	6	12	27	27	
**Echocardiogarphic findings**							
**Major criteria**							
Yes	158	3	7	14	22	25	0.013
No	85	4	15	22	32	36	
**Vegetation**							0.007
Yes	139	4	6	12	22	22	
No	104	3	15	22	30	36	
**Underlying conditions**							
**Diabetes**							
Yes	21	5	5	5	29	29	0.325
No	222	3	10	18	26	30	
**Dialysis**							0.340
Yes	5	25	25	NA	NA	NA	
No	238	3	9	16	25	29	
**Collagen disease**							0.160
Yes	14	7	24	49	49	49	
No	229	3	9	15	24	28	
**Malignancy**							
Yes	11	10	10	40	NA	NA	0.383
No	232	3	10	16	25	28	
**Intravenous drug use**							
Yes	19	0	0	NA	NA	NA	0.164
No	224	4	10	17	26	30	
**Alcohol abuse**							
Yes	22	9	15	15	25	NA	0.660
No	221	3	9	16	25	29	
**Heart failure**††							
Yes	108	6	15	23	28	31	0.110
No	135	2	5	11	23	27	
**Neurological complications**††							
Yes	60	3	11	18	27	27	0.930
No	183	3	9	16	25	30	
**Peripheral emboli**††							0.175
Yes	69	3	8	14	20	20	
No	174	3	10	17	27	32	
**Early surgery (in-hospital)**							
Yes	67	0	5	7	14	18	0.029
No	176	5	12	20	30	34	
**Mode of treatment**††							0.026
Surgical	72	0	4	9	15	19	
Conservative	171	5	12	20	30	34	
**Indications for in-hospital surgery**							
Congestive heart failure	35	0	3	7	22	29	0.269*
Valvular regurgitation without heart failure	18	0	6	6	6	6	
Dehiscence of prosthetic valve without heart failure	8	0	13	13	13	13	
Repeated emboli	3	0	0	0	0	0	
Intractable infection	3	0	0	0	0	0	
**Erythrocyte sedimentation**‡,**§**							
≥ 50 mm/h	75	4	14	23	29	33	0.527
< 50 mm/h	74	1	6	13	30	34	
**White blood cell count**‡,**§S**							
≥ 10 × 10^9^/l	119	3	10	17	30	30	0.781
< 10 × 10^9^/l	98	4	9	17	24	31	
**Serum C-reactive protein**‡,¶							
≥ 100 mg/l	100	3	7	10	13	13	0.080
< 100 mg/l	105	4	12	16	28	39	
**Serum creatinine value**‡,¶P							
≥ 100 μmol/l	76	5	12	24	42	60	0.001
< 100 μmol/l	142	1	7	13	18	18	

*p values for overall group differences; †NA = non-applicable, the follow-up did not reach the time point for any patient of the group; ††within 3 months of admission; ‡on admission; §data available for 149 episodes; §Sdata available for 217 episodes; ¶data available for 205 episodes; ¶¶ data available for 218 episodes

### Long-term overall outcome

**Figure 1:**
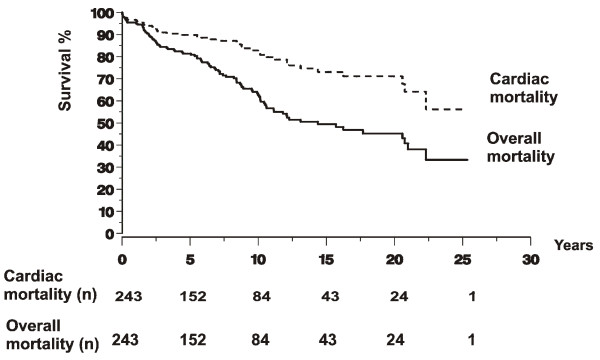
**Kaplan-Meier curves of the overall mortality and cardiac mortality for 243 episodes of infective endocarditis in 226 patients, who survived longer than 1 year after the initial episode.** The numbers at different time points refer to the number of patients being followed up and at risk for event at the beginning of the time period. Zero on the time access indicates 1 year.

**Figure 2:**
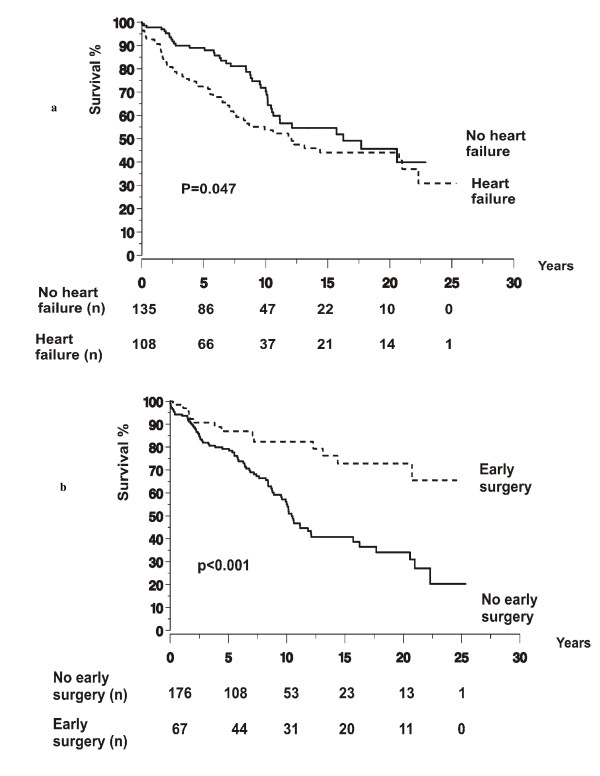
**Long-term outcome was analysed for 243 episodes of infective endocarditis in 226 patients, who survived longer than 1 year after the initial episode.** Kaplan-Meier curves of the overall survival for patients with and without heart failure during 3 months of admission (a), and for those undergoing and not undergoing early (in-hospital) surgery (b). The numbers at different time points refer to the number of patients being followed up and at risk for event at the beginning of the following time period. Zero on the time access indicates 1 year.

Among the 1-year survivors, the long-term overall survival was 95%, 82%, 66%, 51%, and 45% at 2, 5, 10, 15, and 20 years (Figure 1). The association between the survival and various clinical characteristics during the initial episode of IE is presented in Table 1. In univariate analysis, an age ≥ 65 years when contracting the disease was significantly associated with higher mortality, as was malignancy, collagen disease, chronic dialysis, or alcohol abuse as underlying conditions. Mortality was significantly higher for the patients who developed heart failure within 3 months of the initial admission than for those with no heart failure (Figure 2a). Significant differences in mortality were observed between the patients with different predisposing cardiac conditions, the mortality being highest for the patients with congenital heart disease and lowest for the patients with bicuspid aortic valve. Mortality was significantly lower for the patients with IVDU than for those with no IVDU. The long-term survival was significantly better for the patients who underwent early (in-hospital) surgery than for those who did not (Figure 2b). Survival was significantly better for the patients who were treated both surgically and conservatively within 3 months than for those who were treated only conservatively. There were significant differences in survival between the patients who underwent surgery on different indications, the survival being lowest for those operated on for heart failure.

Data on the serum CRP value, ESR, WBC count, and serum creatinine value on admission was available in 205, 149, 217, and 218 episodes of endocarditis, respectively. Higher survival rates were significantly associated with ESR value < 50 mm/h, or serum creatinine value < 100 μmol/l on admission.

In age and sex adjusted multivariate analysis, significant predictors for mortality were heart failure within 3 months of admission (HR 1.97, 95% CI 1.27 to 3.06; p = 0.003) and collagen disease (HR 2.54, 95% CI 1.25 to 5.19; p = 0.010) or alcohol abuse (HR 2.39, 95% CI 1.30 to 4.40; p = 0.005) as underlying conditions. Early surgery was significantly associated with lower overall mortality rates (HR 0.31, 95% CI 0.17 to 0.58; p < 0.001). Also when recurrent episodes of IE were excluded from the analyses, heart failure (HR 1.73, 95% CI 1.10 to 2.72; p = 0.018), collagen disease (HR 2.67, 95% CI 1.30 to 5.49; p = 0.008), and alcohol abuse (HR 2.80, 95% CI 1.51 to 5.19; p = 0.001) remained significant risk factors for mortality, and early surgery was significantly associated with lower overall mortality rates (HR 0.32, 95% CI 0.17 to 0.59; p < 0.001).

### Long-term cardiac mortality

Among the 1-year survivors, the long-term cardiac mortality was 3%, 10%, 16%, 25%, and 29% at 2, 5, 10, 15, and 20 years (Figure 1). In univariate analysis, cardiac mortality was significantly associated with an age ≥ 65 years when contracting the disease. There were significant differences in the long-term cardiac mortality between various causative agents of IE, the mortality being highest for *Enterococcus faecalis*, as well as between various predisposing cardiac conditions, the mortality being highest for acquired valvular disease. The long-term cardiac mortality was significantly lower for the patients who underwent early surgery than for those who did not. Mortality was significantly lower for the patients who were treated both surgically and conservatively within 3 months than for those who were treated only conservatively. The long-term cardiac mortality was significantly higher for the patients with serum creatinine value ≥ 100 μmol/l on admission than for those with lower serum creatinine values. The long-term cardiac mortality was significantly lower for the patients who had a major criterion or vegetation on echocardiography than for those who had no such findings on echocardiography.

In age and sex adjusted multivariate analysis, heart failure during 3 months of admission was significantly associated with high long-term cardiac mortality rates (HR 1.99, 95% CI 1.06 to 3.73; p = 0.032), whereas the presence of a vegetation of echocardiography was significantly associated with low long-term cardiac mortality rates (HR 0.40, 95% CI 0.21 to 0.76; p = 0.005). When recurrent episodes were excluded from the analyses, patients with heart failure had higher cardiac mortality rates, but the association did not reach statistical significance (HR 1.81, 95% CI 0.95 to 3.44; p = 0.070). Yet, vegetation remained as a factor significantly associated with lower cardiac mortality rates (HR 0.44, 95% CI 0.23 to 0.84; p = 0.012).

### Late valve surgery

Of all 326 episodes of IE in 303 patients treated between 1980 and 2004, valve surgery during the follow-up was performed in 126. Fifteen patients underwent 2 valve operations and 1 patient 3 valve operations. In 17 patients, the first valve operation was performed > 1 year after the initial admission. Eight of them were operated on between 1 and 2 years, 5 patients between 2 and 5 years, 3 patients between 5 and 9 years, and 1 patient 16 years after the index episode of IE. In addition, 3 patients undergoing the first valve surgery during the initial hospitalisation underwent a reoperation more than 1 year (range 4 to 7 years) later. Thus, altogether 20 patients underwent late valve surgery. The indications for late valve surgery were: valvular regurgitation without heart failure (n = 15), dehiscence of prosthetic valve (n = 3), valvular stenosis (n = 1), and heart failure (n = 1).

Patients who underwent late valve surgery had significantly lower overall mortality rates than those who did not (HR 0.36, 95% CI 0.15 to 0.90; p = 0.029), but the cardiac mortality rates were not significantly different (HR 0.50, 95% CI 0.15 to 1.62; p = 0.247).

Based on univariate analyses, patients with neurological complications within 3 months of admission required late valve surgery significantly less common than those with no neurological complications (p= 0.032). In age and sex adjusted multivariate analysis, no clinical factor during the initial hospitalisation was associated with the requirement of late valve surgery.

Patients with recurrences did not require late valve surgery more commonly that those with no recurrences (HR 1.04, 95% CI 0.24 to 4.63; p = 0.955).

### Survival free of complications

**Figure 3:**
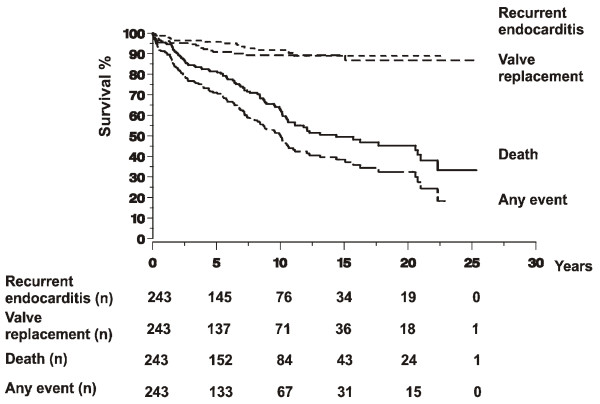
**Kaplan-Meier curves of survival free of recurrent endocarditis, valve replacement, and death after an episode of infective endocarditis.** The numbers at different time points refer to the number of patients being followed up and at risk for event at the beginning of the following time period. Zero on the time access indicates 1 year.

Among the 1-year survivors, survival free of complications (death, valve replacement, recurrence) after an episode of IE is shown in Figure 3.

### Repeated episodes of IE

Of all 303 patients treated between 1980 and 2004, 20 had more than 1 episode of IE including 17 patients with 1 recurrence of IE, 2 patients with 2 recurrences of IE, and 1 patient with 1 recurrence and 1 relapse of IE. The relapse was a PVE caused by a *Staphylococcus aureus *strain genotypically identical with the strain causing native valve IE in the same patient 3 months earlier. Of the 23 repeated episodes of IE, prosthetic valves were involved in 16.

#### 1-year survivors

A total of 16 recurrences occurred among the 1-year survivors. In univariate analysis, the only factor during the index episode of IE significantly associated with recurrences in these patients was chronic dialysis (p < 0.001). This association remained significant also in multivariate analysis (HR 20.73, 95% CI 4.08 to 105.40; p < 0.001). Patients with recurrent episodes of IE had significantly lower overall mortality rates than those with no recurrences (HR 0.09, 95% CI 0.01 to 0.64; p = 0.017).

#### All patients

In univariate analysis, the factors during the index episode of IE significantly associated with recurrences in all 303 patients with 23 recurrences were age < 65 years (p = 0.016) and diabetes (p = 0.022), chronic dialysis (p = 0.002) or IVDU (p < 0.001) as underlying conditions. Also in age and sex adjusted multivariate analysis, chronic dialysis (HR 12.56, 95% CI 2.54. to 62.24; p = 0.002), IVDU (HR 5.49, 95% CI 1.84 to 16.43; p = 0.002) and diabetes (HR 3.71, 95% CI 1.30 to 10.62; p = 0.015) remained significant. Patients with recurrent episodes of IE had significantly lower overall mortality rates than those with no recurrences (HR 0.05, 95% CI 0.01 to 0.33; p = 0.002).

## Discussion

In this study, we analysed the long-term outcome of the patients treated for IE in a Finnish teaching hospital during a period of 25 years. Among the 1-year survivors, the overall survival was 66% at 10 years, 51% at 15 years and 45% at 20 years. These figures are comparable with the results of previous studies [12,15], although we are not aware of any earlier study in which the long-term survival has been analysed in an identical manner. Collectively, these data illustrate that despite major diagnostic and therapeutic advances, the mortality associated with IE remains high. At 10 years of follow-up, as many 51% of our whole patient population and 34% of the 1-year survivors had died. Admittedly, a few studies have been published reporting somewhat higher survival rates [13].

One of the main purposes of the present study was to define which clinical characteristics or complications of IE during the initial phase of endocarditis would be associated with an adverse long-term outcome of the patients. Consistent with previous studies [12,14], older age at the time of contracting the disease predicted an adverse long-term outcome. Chronic dialysis as an underlying condition predicted a poor overall long-term prognosis, as did alcohol abuse and collagen disease. In the patients with collagen disease, the severe nature of the underlying disease may have contributed to high mortality. Of all 9 patients with collagen disease who died during the follow-up, 3 needed chronic dialysis, 1 had nephrotic syndrome and in 3 patients, the cause of death was a malignancy. On the other hand, the survival was significantly better for the patients with IVDU than for those with no IVDU, but all of the patients with IVDU were young and most of them had a tricuspid valve disease with no mortality. Moreover, the follow-up time of these patients was short, since the first IVDU-associated case of IE occurred in our hospital in 1996.

Among the complications of IE, heart failure during the first 3 months of admission was significantly associated with long-term overall mortality and cardiac mortality. This corroborates other studies, which have found that heart failure is a major risk factor for long-term mortality [15]. Also among the surgically treated patients, the overall survival was lowest for those who were operated on for heart failure, underscoring the importance of heart failure as a poor long-term prognostic sign.

Neurological manifestations and peripheral emboli usually develop early in the course of endocarditis [16,22-24]. We have previously shown that in this patient population, the occurrence of neurological complications or peripheral emboli was significantly associated with short-term mortality as well as with mortality for up to 1 year of the admission [16]. In contrast, the results of the present study show that the long-term survival was practically similar for the patients with or without neurological manifestations or peripheral emboli during the acute phase of their illness (Table 1). This finding is not surprising, since once the patient has solved such an acute complication, it seems logical that it no longer influences the long-term prognosis.

In our series, early surgery was performed in 27.3% (89) of all 326 episodes [16]. Evaluation of the role of cardiac surgery on the prognosis of the patients with IE is a complex issue. Although early surgery is evidently life saving for certain groups of patients, it does not necessarily decrease the total mortality, since more and more critically ill patients are being treated surgically. This may be one of the reasons why the results regarding the role of early surgery as a prognostic factor have been conflicting. For example, when studying 280 patients treated in their unit between 1970 and 1982, Malquarty et al. [25] did not find any significant difference in the survival rate at 5 years between the surgical and non-surgical treatment groups. Neither did Tornos et al. [8] find any association between early surgery and a better survival rate during a 15-year follow-up among 140 patients hospitalised between 1975 and 1990. On the other hand, according to Bishara et al. [26], early surgical intenvention compared with medical therapy alone was associated with increased long-term survival rates in patients with IE treated between 1987 though 1996, primarily when IE was caused by *S. aureus. *Similarly, long-term survival was predicted by early surgical treatment in 212 patients with IE treated between 1980 and 1995 in a series described by Netzer at al. [15]. Moreover, Castillo et al. [13] have reported that a high early surgery rate was related to good long-term results in 138 cases of IE hospitalised between 1987 and 1997. Our results corroborate these findings: early surgery led to significantly lower overall and cardiac mortality rates in these 1-year survivors, although in multivariate analysis, the association was not significant regarding the long-term cardiac mortality. Thus, the results of the present study support the contention that a successful and correctly focused early valve surgery may decrease mortality due to IE. This could be accomplished by a careful selection of appropriate patients to be treated surgically.

The prognosis of the patients surviving the initial phase of endocarditis is considered to be due to 3 main factors: the development of heart failure, the risk of recurrences and the need for later valve replacement. In our patients, repeated episodes of IE were not common, as 20 (6.6%) of all 303 patients had recurrences and only 1 patient (0.3%) had a relapse. The rate of recurrences concurs with other published series, in which the rate of recurrences has varied between 3.9 and 16% [12,14,27-29], but the rate of relapses was here somewhat lower than the 1.1 to 3.3% relapse rate reported in earlier studies [8,14,30]. Chronic dialysis, diabetes and IVDU were shown to be risk factors for the development of recurrences when analysed for all 303 patients treated in our hospital between 1980 and 2004. IVDU has been shown to be a risk factor also in some other [29], but not all [14], previous studies. Although recurrent endocarditis is considered to be a risk factor for death [14], the development of a recurrent episode of IE did not confer a poor prognosis in our patients; in direct contrast, patients with recurrences had a better long-term overall outcome than those with no recurrences. At least a partial explanation for this could be that IVDU was a risk factor for the recurrences of IE. The patients with IVDU had a significantly lower mortality than those with no drug use, evidently due to the fact that they commonly had tricuspid valve IE with low mortality.

Late surgery was required in 17 (7.0%) of the 243 episodes. None of the clinical parameters during the initial episode of IE were found to be associated with the requirement for late valve surgery. The requirement for late surgery was not increased even in the patients with recurrent episodes of IE. Also this could be partly explained by the positive association between the development of recurrences and IVDU.

Survival free of complications decreased with time. Although the estimate for 20-year survival was 45%, the estimate for 20-year survival free of complications, i.e. recurrent endocarditis or valve replacement, was only a little more than 30%.

## Conclusion

In our patients, heart failure within 3 months of the admission for the index episode of IE was the complication, which significantly predicted a poor long-term outcome. Patients who underwent surgery during the initial hospitalisation for IE faired significantly better than those who did not. Corroborating earlier findings, these results support the contention that a successful and correctly focused early valve surgery may decrease mortality due to IE. This could be accomplished by a careful selection of appropriate patients to be treated surgically. It must be admitted that the heterogeneity of the patient population included in this study may be one limitation when applying our results in the daily clinical practice. An analysis of long-term prognostic factors in some defined subgroups of our whole study population may provide further valuable information in the future.

## Competing interests

The author(s) declare that they have no competing interests.

## Authors' contributions

All of the authors contributed substantially to the study. MH collected the data. MH and PK designed the study and wrote the original version of the manuscript. HH, SH, TS, KM, EE and JN contributed to evaluation of data and provided critical comments for the manuscript. HH and SH performed the statistical analyses. All authors read and approved the final version of the manuscript.

## Pre-publication history

The pre-publication history for this paper can be accessed here:


